# TIRAP in the Mechanism of Inflammation

**DOI:** 10.3389/fimmu.2021.697588

**Published:** 2021-07-08

**Authors:** Sajjan Rajpoot, Kishore K. Wary, Rachel Ibbott, Dongfang Liu, Uzma Saqib, Teresa L. M. Thurston, Mirza S. Baig

**Affiliations:** ^1^ Department of Biosciences and Biomedical Engineering (BSBE), Indian Institute of Technology Indore (IITI), Indore, India; ^2^ Department of Pharmacology and Regenerative Medicine, The University of Illinois at Chicago, Chicago, IL, United States; ^3^ MRC Centre for Molecular Bacteriology and Infection, Imperial College London, London, United Kingdom; ^4^ Department of Pathology, Immunology and Laboratory Medicine, Rutgers University-New Jersey Medical School, Newark, NJ, United States; ^5^ School of Graduate Studies, Rutgers Biomedical and Health Sciences, Newark, NJ, United States; ^6^ Center for Immunity and Inflammation, New Jersey Medical School, Rutgers-The State University of New Jersey, Newark, NJ, United States; ^7^ Discipline of Chemistry, Indian Institute of Technology Indore (IITI), Indore, India

**Keywords:** inflammation, TLR signaling, inflammatory disease, TIRAP (TIR domain-containing adaptor protein), protein-protein interaction (PPI)

## Abstract

The Toll-interleukin-1 Receptor (TIR) domain-containing adaptor protein (TIRAP) represents a key intracellular signalling molecule regulating diverse immune responses. Its capacity to function as an adaptor molecule has been widely investigated in relation to Toll-like Receptor (TLR)-mediated innate immune signalling. Since the discovery of TIRAP in 2001, initial studies were mainly focused on its role as an adaptor protein that couples Myeloid differentiation factor 88 (MyD88) with TLRs, to activate MyD88-dependent TLRs signalling. Subsequent studies delineated TIRAP’s role as a transducer of signalling events through its interaction with non-TLR signalling mediators. Indeed, the ability of TIRAP to interact with an array of intracellular signalling mediators suggests its central role in various immune responses. Therefore, continued studies that elucidate the molecular basis of various TIRAP-protein interactions and how they affect the signalling magnitude, should provide key information on the inflammatory disease mechanisms. This review summarizes the TIRAP recruitment to activated receptors and discusses the mechanism of interactions in relation to the signalling that precede acute and chronic inflammatory diseases. Furthermore, we highlighted the significance of TIRAP-TIR domain containing binding sites for several intracellular inflammatory signalling molecules. Collectively, we discuss the importance of the TIR domain in TIRAP as a key interface involved in protein interactions which could hence serve as a therapeutic target to dampen the extent of acute and chronic inflammatory conditions.

## Introduction

TIRAP, also known as MyD88-adaptor Like (MAL), is an adapter molecule associated with receptor-mediated activation of host immune signaling (1, 2). The innate immune system recognizes microbial pathogens through receptors, including Toll-like receptors (TLRs), which identify pathogen-associated molecular patterns (PAMP) (3, 4). Upon ligation of most TLRs with their respective ligands (PAMP), MyD88 is recruited directly to the cell surface receptor intracellular domain. In the case of TLR2 and TLR4, Myd88 recruitment is indirect and occurs *via* TIRAP. This event nucleates the formation of a large signaling complex called the “Myddosome” (5–7). The resulting downstream signaling events culminate in the activation of several transcription factors that include nuclear factor κB (NF-κB) and activated protein 1 (AP1) (6, 8–10). The activation of TLR-mediated signaling pathways is critical in driving the induction of proinflammatory cytokines by immune cells and controlling host cell survival. In this way, the cell is reprogrammed to a state that helps mitigate infection. Regulated TLR activation is required for host defense activities, yet unwanted amplification of proinflammatory cytokines is detrimental to the host. Furthermore, inappropriate receptor activation can propagate the development of autoimmune diseases in individuals harboring genetic risk factors. Therefore, it is critical to understand the TLR- and TIRAP-mediated signaling mechanisms and how these pathways are regulated.

**Figure 1:**
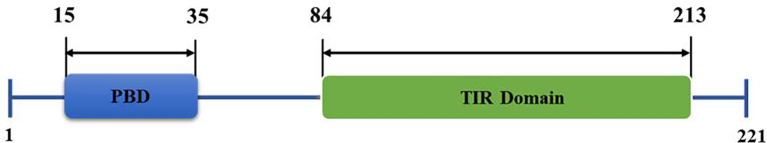
Structural organization of Toll/interleukin 1 receptor (TIR) domain-containing adaptor protein (TIRAP) domains. The amino acid position of an N-terminal phosphatidylinositol (PI) binding domain (PBD) and a C-terminal Toll-like receptor (TIR) domain are as shown.

TIRAP consists of 221 amino acids, constituting two main domains (**Figure 1**); a phosphatidylinositol 4,5-bisphosphate (PIP_2_) binding domain (PBD), which is responsible for targeting TIRAP to discrete regions of the plasma membrane upon Phosphatidylinositol 4-Phosphate 5-Kinase (PIP5Kα)-mediated production of PIP_2_ (11, 12) and a TIR domain, which is mainly involved in protein-protein interactions with numerous inflammatory-related proteins (6, 13, 14).

**Figure 2:**
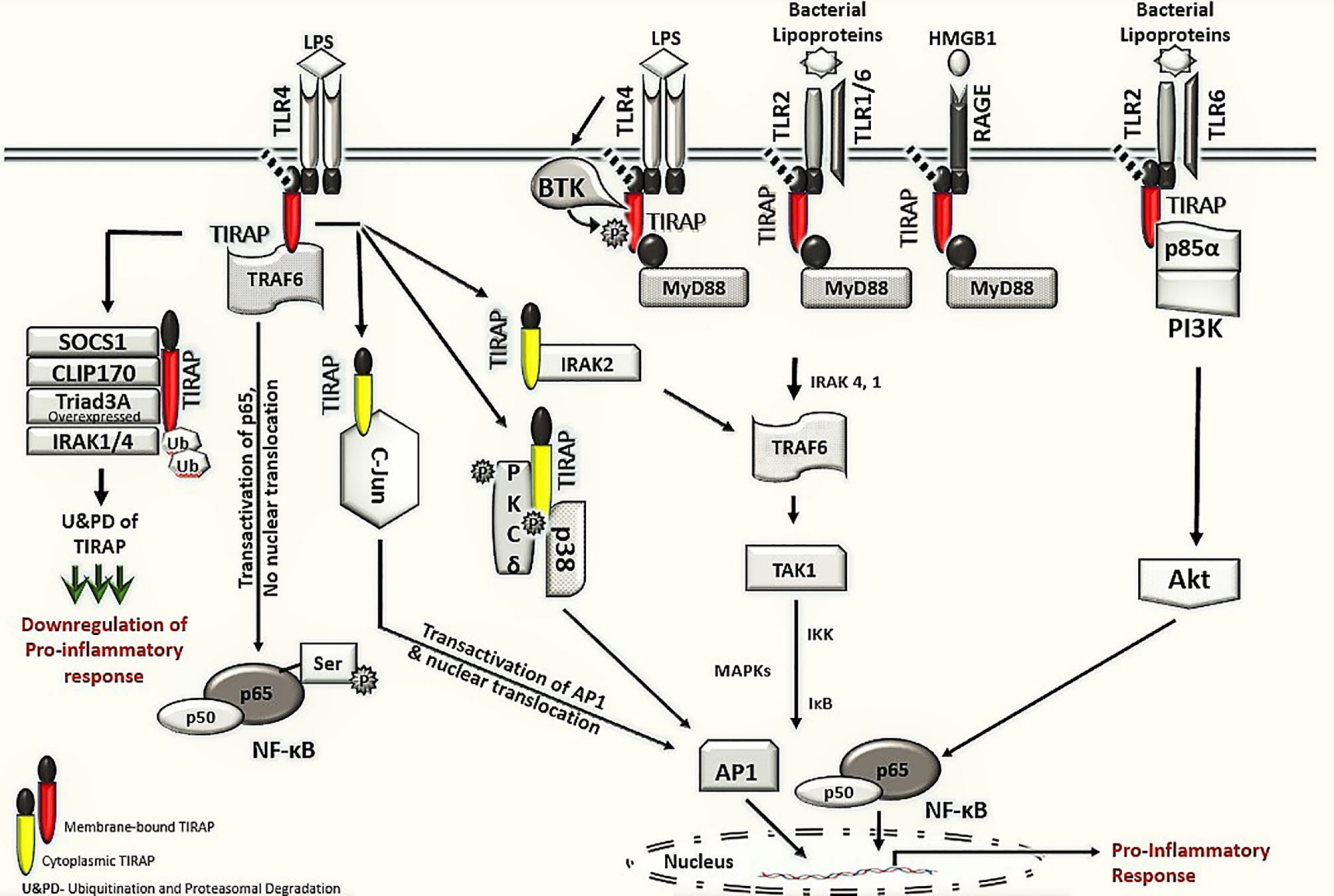
TIRAP interacting machinery in the activation of inflammatory signaling. The membrane localized TIRAP initiates the downstream signaling via its interaction with upstream membrane bound receptors TLR4, TLR2 and RAGE as well as with membrane localized kinases BTK, p85α subunit of PI3K and BCAP. In TLR4 mediated signaling, the membrane localized TIRAP also interacts with TRAF6 for transactivation of the p65 in NF-κB pathway. The downstream inflammatory signaling *via* cytoplasmic TIRAP involves an interaction with protein kinase PKCδ and p38 MAPK as well as with IRAK2 and AP-1 subunit c-Jun, respectively. The negative regulators of TIRAP protein including SOCS1, CLIP170, IRAK1/4 and Triad3A are involved in interactions with TIRAP for its ubiquitination and proteasomal degradation (U&PD) and hence downregulation in inflammatory response mediated *via* TIRAP. TLR- Toll -interleukin-1 receptor; TIRAP- Toll-interleukin-1 Receptor (TIR) domain-containing adaptor protein; MyD88- Myeloid differentiation factor 88; RAGE- Receptor for advanced glycation end-products; BTK- Bruton’s tyrosine kinase; PI3K- phosphoinositide 3-kinase; BCAP- B-cell adaptor for phosphoinositide 3-kinase; TRAF6, Tumor necrosis factor receptor (TNFR)-associated factor 6; NF-κB, Nuclear Factor kappa-light-chain-enhancer of activated B cells; PKCδ, protein kinase c delta; p38 MAPK, p38 mitogen-activated protein kinase; AP-1, Activator protein 1; SOCS1, Suppressor of cytokine signaling 1; CLIP170, Cytoplasmic linker protein 170; IRAK, interleukin-1 receptor-associated kinase; IKK, IκB kinase; and IκB, nuclear factor of kappa light polypeptide gene enhancer in B-cells inhibitor.

This review discusses the diverse TIRAP protein interacting partners (summarized in **Table 1**) and the significance of these during inflammatory signaling pathways and the onset of inflammatory diseases. First, we summarized the mechanism of recruitment of TIRAP; thereafter, we discuss the downstream interactions that mediate inflammatory signaling. In that respect, the **Figure 2** is mainly summarized to provide the insight of inflammatory signaling pathways mediated *via* multiple TIRAP interactions eventually leading to the activation of major transcription factors NF-κB and AP-1 and hence expression of proinflammatory cytokines (2, 9, 16, 20, 26, 29, 30, 32, 40, 41). On the other hand, the negative regulators of TIRAP interacting proteins are discussed and represented for their known roles (18, 22, 23, 35, 42).

**Table 1:** The significance of TIRAP interaction with the partner proteins in the cellular process.

Sr No.	Interactions	Nature of Response	Role of the Interaction in Inflammatory Pathways	References
**1**	TIRAP-TLR4	Pro-inflammatory response	Interaction transduces downstream signaling *via* MyD88 dependent pathway. It bridges MyD88 to TLR4 and hence activates NF-κB and AP-1 nucleus translocation and cytokines expression.	(2, 6, 8, 15)
**2**	TIRAP-TLR2	Proinflammatory response	As in TLR4, this interaction also bridges TRL2 to MyD88 and leads to the production of the inflammatory cytokines *via* the activation of NF-κB, AP-1, and MyD88-independent PI3k-Akt pathway.	(6, 16, 17)
**3**	TIRAP-MyD88	Proinflammatory response	In MyD88 dependent-pathway, the TIRAP acts as the bridging protein to bring the MyD88 to TLR4/2 inflammatory signaling.	(2, 5, 6, 8, 14, 15)
**4**	TIRAP-CLIP170	Inhibitory	TIRAP interacts with CLIP170 which leads to its ubiquitination and promotes proteasomal degradation resulting in reduced NF-κB and AP-1 response.	(18)
**5**	TIRAP-RAGE	Proinflammatory response	The stimulated RAGE receptor, similar to TLR4, binds TIRAP to activate MyD88 dependent NF-κB and AP-1 inflammatory pathways.	(19)
**6**	TIRAP-TRAF6	Proinflammatory response	In stimulated TLR4 pathway, the interaction leads to transactivation of p65 by its direct phosphorylation at serine-536 (p-S536) residue.	(20)
**7**	TIRAP-p85α	Proinflammatory response	TIRAP interacts with PI3K subunit p85α in response to the TLR2-TLR1/6 heterodimer response to activate the PI3K-Akt pathway.	(16, 21)
**8**	TIRAP-Triad3A	Inhibitory	In overexpressed condition, Triad3A, an E3 ubiquitin ligase interacts with TIRAP for its U&PD	(22)
**9**	TIRAP-SOCS1	Inhibitory	SOCS1 interacts with BTK phosphorylated TIRAP to negatively regulate it by U&PD	(23–25)
**10**	TIRAP-BTK	Proinflammatory response	In stimulated TLR4/2 pathways, the interaction with BTK is crucial for TIRAP activation *via* its tyrosine phosphorylation at TIR domain leading to downstream NF-κB and AP-1 activation	(17, 26–28)
**11**	TIRAP-PKCδ	Proinflammatory response	The interaction leads phosphorylation and activation of TIRAP which promotes the downstream p38 MAPK and NF-κB response in stimulated macrophages.	(27, 29, 30)
**12**	TIRAP-p38 MAPK	Proinflammatory response	The interaction leads to the activation of AP-1 and enhanced pro-inflammatory cytokine response in stimulated macrophages	(30, 31)
**13**	TIRAP-c-Jun	Proinflammatory response	In stimulated TLR4 pathway, this interaction leads to the transactivation of c-Jun and its nucleus translocation for proinflammatory genes expression	(32)
**14**	TIRAP-IRAK2	Proinflammatory response	In TLR4/2 pathway, this interaction activates NF-κB independent of MyD88 *via* TRAF6 and TAK protein leading to increased pro-inflammatory response	(2, 26)
**15**	TIRAP-Caspase1	Inhibitory	Caspase1 interaction leads to activation of TIRAP to increase NF-κB transcriptional activity. In a contrast study, D198E, however, function normally and hence demand more investigation.	(33, 34)
**16**	TIRAP-IRAK1/4	Inhibitory	In stimulated TLR4/2 pathways, TIR domain mediated interaction leads to the phosphorylation at T28 and other probable sites in TIRAP leading to its U&PD	(35)
**17**	TIRAP-BCAP	Inhibitory	This interaction negatively regulates TLR signaling. Dimeric BCAP interfere with the TLR-PI3K signalling and associates with TIR domain of TIRAP to negatively regulate the inflammatory response.	(36–39)

U&PD-Ubiquitination and Proteasomal degradation.

## TIRAP and Bruton’s Tyrosine Kinase (BTK)

For TIRAP to carry out its function as a TLR adaptor protein in inflammatory signaling, it must be recruited to the membranes’ activated receptors. This occurs upon binding to PIP_2_ before its interaction with TLRs (12). Subsequent interaction with TLRs’ TIR domain is facilitated by tyrosine phosphorylation of selected TLRs and their adaptor molecules by several tyrosine kinases, including Bruton’s tyrosine kinase (BTK), Src, Lyn, Syk, etc (43, 44). For example, phosphorylation of the cytoplasmic tail of the TLR4 TIR domain at Y674 and Y680 and phosphorylation of TIRAP tyrosine residues at positions 86, 106, 159, and 187 by BTK are required for TLR4-TIRAP-MyD88 interaction and activation of NF-κB and MAPK (mitogen-activated protein kinase) signaling leading to proinflammatory responses (**Figure 2**) (17, 26, 45, 46). BTK interaction with TIRAP and its tyrosine phosphorylation in the TIR domain is crucial for TIRAP to function. Six conserved tyrosine (Y) residues in the TIR domain of human TIRAP are the potential phosphor-acceptor site. The four residues Y86, Y106, Y159 and Y187 in TIRAP are experimentally identified as the sites for BTK mediated phosphorylation. Meanwhile, the mutation of these tyrosine residues either with alanine or phenylalanine describes them as the crucial sites for BTK mediated phosphorylation, as after stimulation, the mutation impaired the BTK association with TIRAP, similar to the previously reported P125H variant of TIRAP. Also, these mutations play a dominant negative role and impair NF-κB activation (13, 17, 26). Several studies hence concluded the significance of tyrosine sites on TIRAP in the TIR domain for association with BTK immediately on LPS stimulation. However, it is important to have a detailed understanding of the mechanism of TIRAP-BTK interaction and the role of tyrosine phosphorylation regulating the downstream signaling. The prerequisite step in the TIRAP phosphorylation by BTK is its activation itself. In stimulated macrophages, src kinase is reported to activate BTK as well as PKCδ (protein kinase C delta) (27–30, 47, 48). Structural analysis of BTK and PKCδ interaction with TIRAP suggests that Y106 of TIRAP is phosphorylated by the action of both BTK and PKCδ (27). Both phosphorylation sites promote the activation of downstream p38-MAPK, NF-κB pathways, and the associated cytokine response, but due to ubiquitin-dependent degradation of phosphorylated TIRAP mediated by SOCS1 (suppressor of cytokine signaling 1), TIRAP tyrosine phosphorylation by BTK is transient (23). With the critical molecules poised to become activated at the membrane, BTK-mediated TIRAP phosphorylation and activation, followed by degradation of phospho-TIRAP, results in a rapid yet balanced inflammatory response, avoiding prolonged TLR4 or TLR2 signaling that would otherwise result in chronic inflammation and associated diseases (23). A recently concluded study on BCR-TLR interplay suggest that the high expression of BTK in TLR signaling leads to the development of pathology in a Btk-dependent model for systemic autoimmune disease (49).

## TIRAP and Receptor for Advanced Glycation End-Products (RAGE)

The Receptor for advanced glycation end-products (RAGE) is a multi-ligand cell membrane receptor implicated in diverse chronic inflammatory states such as cardiovascular disease, cancer, neurodegeneration, and diabetes (50–52). RAGE is activated by diverse damage-associated molecular pattern molecules (DAMPs), which include advanced glycation end products (AGEs), high mobility group box-1 (HMGB1), and S100 proteins (51–53). Upon ligand binding, Protein Kinase C-ζ (PKC-ζ) phosphorylates the cytosolic domain of RAGE on Ser391, mediating interaction with the TIR domain TIRAP (19). As with TLR signaling, TIRAP acts as a bridge to MyD88. In this way, RAGE activation induces MyD88-dependent proinflammatory signaling (19) (**Figure 2**). The soluble RAGE is generated after either the proteolytic cleavage of its transmembrane domain to generate sRAGE or alternative splicing to give endogenous soluble RAGE (esRAGE). These proteins have distinct roles in inflammation and disease (compared to RAGE), with neither capable of inducing signaling upon binding RAGE targets. Instead, sRAGE blocks RAGE signaling effectively and appear to prevent or reduce inflammatory conditions (50, 51, 54). As mutations in the TIR domain of TIRAP result in inhibition of downstream inflammatory signaling and the role of RAGE in inducing a proinflammatory immune response during disease (19). The RAGE signalling plays a vital role in many inflammatory associated diseases (acute lung injury, sepsis, inflammatory bowl disease, atherosclerosis, cancer and other chronic infectious and noninfectious diseases). Many ligands (e.g., HMGB1, s100, etc) activate both RAGE and TLR4 leading to same inflammatory pathway *via* TIRAP interaction, a direct cross talk between both signalling has also been highlighted (55, 56). It should now be explored in detail whether the therapeutic intervention of TIRAP and RAGE represents a means to treat these diseased states. An earlier study on neuronal cells has been reported to disrupt this interaction through decoy RAGE peptide (RAGE-I) targeting TIRAP and abrogates the activation of cdc42, inhibiting cell migration and invasion and protecting cell death (57).

## TIRAP and Phosphatidylinositol 3′-Kinase p85 and B-Cell Adaptor for Phosphoinositide 3-Kinase (BCAP)

The interaction of p85α, a regulatory subunit of phosphoinositide 3-kinase (PI3K) with TIRAP, is a MyD88-independent response of the TLR2 receptor upon stimulation with bacterial lipoproteins (16, 21). This interaction is highly significant in TLR2 and TLR6 heterodimer signaling, resulting in the activation of PI3K-dependent phosphorylation of Akt, PIP_3_ [phosphatidylinositol (3,4,5)P_3_] generation, and polar shape changes of the macrophage (16). Signaling is initiated when TLR2 heterodimers with TLR6 are bound by diacylated lipoprotein ligands, or when TLR1/2 heterodimers are bound by triacylated lipoproteins (58). Ligand binding induces conformational changes within the receptor that bring their TIR domains close to downstream signal transduction (1, 59, 60). Upon diacylated lipoprotein stimulation of TLR2/6, TIRAP, but not MyD88, is essential for PI3K activity and NF-κB activation (16). Upon stimulation of macrophages, TIRAP interacts with p85α, and these proteins colocalize at the plasma membrane (16). Whereas MyD88 is not required for interaction of TIRAP with p85α, the efficiency of PI3K activity, and therefore downstream activation of Akt and NF-κB, as well as macrophage polarization, becomes delayed in MyD88 deficient cells (16). This suggests that the TIRAP interaction with p85α is direct but that MyD88 might accelerate the kinetics of Akt phosphorylation and PIP_3_ generation.

MALP-2 induced activation of the TLR2/6 pathway in THP-1 cells also induces the interaction of TIRAP with p85α, and this is essential for the induction of Heme Oxygenase-1 (HO-1) *via* Akt phosphorylation and Nrf2 activation (21). Similar to the above study, this study also reported that MyD88 deficiency resulted in decreased Akt phosphorylation but at earlier time points post-activation (60 minutes). HO-1 expression upon MALP-2 stimulation also involves c-Src and BTK, which are other binding partners of TIRAP. These proteins are likely to form a complex consisting of c-Src, BTK, TIRAP, and p85α upon stimulation, and together, they represent a potentially important target for pharmacotherapy during various chronic inflammatory diseases.

In contrast, a multimodular protein, B-cell adaptor for phosphoinositide 3-kinase (PI3K) (BCAP) has been reported to negatively regulate the TLR4/2-PI3K signalling, suggesting its association with TLR and downstream TIR domain of TIRAP, leading to its recruitment to TLR signalasome by TIR-TIR interactions (36–38). BCAP also interacts with p85α suggesting its role in regulating the downstream signalling (36). BCAP is a dimeric protein and its oligomerization depends on its ANK (ankyrin repeat) and DBB (Dof/BANK1/BCAP) domains. A recently concluded study clearly defines the importance of DBB domain in dimerization and its role in TIRAP-BCAP interaction (39). The monomeric BCAP, though fails to negatively regulate the TLR signaling suggesting that only domain dimerization drives the negative response. The TIRAP TIR domain is reported to assemble to form filament complex *in vitro*, an event critical for signal transduction. Such a filament could be disrupted by dimeric, and not monomeric, BCAP. Another angle suggest BCAP phosphoinositide metabolism, which cleaves PIP2 to DAG and IP3 and hence deprives TIRAP for its membrane anchor required for TLR signaling (39). Overall, the BCAP association mainly with TIRAP and p85α provides novel directions for regulatory pathways in inflammation.

## TIRAP and Protein Kinase C-δ (PKCδ)

As mentioned above, PKCδ represents an interacting partner of TIRAP that is required for TLR2- and TLR4-induced activation of p38 MAPK, NF-κB and proinflammatory cytokine expression (29, 30). Mice harboring PKCδ-deficiency reduces the bacterial killing function of macrophages, showing decreased NO, ROS, and H2O2, resulting in hyper-susceptible animals to infection with mycobacterium tuberculosis with increased mortality (61). Mechanistically, PKCδ constitutively interacts with and phosphorylates Y106 in the TIR domain of TIRAP as described above, and BTK phosphorylates TIRAP at Y106, as well as Y86 (27). Accordingly, Baig et al., 2017 reported a novel heterotrimeric complex consisting of TIRAP, PKCδ, and p38 MAPK required for AP1-mediated inflammatory responses in macrophages in response to LPS stimulation (30). Thus, TIRAP activity is regulated in response to receptor stimulation. In this situation, TIRAP was not required for the phosphorylation of PKCδ but was required for cytokine production (30). In summary, PKCδ activation is likely required downstream of TIRAP; however, it remains unclear how PKCδ activation is regulated in macrophages upon LPS stimulation.

## TIRAP and Cytoplasmic Linker Protein 170 (CLIP170)

Cytoplasmic linker protein 170 (CLIP170), containing two conserved cytoskeleton-associated protein Glycine rich (CAP-Gly) domains and two tandem repeats of zinc knuckle motifs (also called CCHC zinc fingers), is a multifunctional protein that binds to and regulates the dynamics of the growing plus end of microtubules (62–64). In addition, CLIP170 acts as a negative regulator of TLR4 by inducing TIRAP ubiquitination (both mono and polyubiquitination) and promoting its proteasomal degradation. Accordingly, this activity of CLIP170 decreased downstream signaling activity, including reduced NF-κB MAPK activity. Further, the activity of CLIP170 is specific to TIRAP, with MyD88 levels unaltered upon overexpression of CLIP170 (18). Analogous to many negative regulators, CLIP170 expression is induced upon stimulation with LPS. Interestingly, the pathogenic bacteria *Brucella* species viz. *Brucella melitensis, Brucella abortus*, and *Brucella ovis* inhibit TIRAP activity by encoding a TIR domain-containing effector protein TcpB, also called Btp1 (65). TcpB, like TIRAP, shares phosphoinositide binding properties and resemblance in its TIR domain; however, functionally, TcpB impairs TLR-4 and TLR-2 induced NF-κB activation inflammatory responses (18, 65). Mechanistically, TcpB interacts with TIRAP and promotes CLIP170-dependent polyubiquitination and subsequent proteasomal degradation of TIRAP (18). CLIP170 is also of therapeutic interest; Pregnenolone, a steroid hormone precursor, suppresses TLR4 and TLR2 mediated inflammation by promoting CLIP170-mediated ubiquitination and degradation of TIRAP (66).

## TIRAP and c-Jun

Delivery of bacterial-derived pathogenic LPS to TLR4 has been shown to activate AP-1 through a series of phosphorylation events on serine/threonine residues mediated by upstream MAPKs (extracellular signal-regulated kinases; ERK, c-Jun N-terminal kinase; JNK, and p38 mitogen-activated protein kinases; p38 kinase) (67). For example, c-Jun, which contains a transactivation domain, is phosphorylated at Ser 63 and 73 by JNKs, resulting in its activation (67). c-Jun can form homo and heterodimers with other AP-1 family members, including c-Fos or ATF (Activating transcription factor) to make transcriptionally active complexes (68–70); (71). These transcriptionally active dimeric components of AP-1 now control the activation of critical proinflammatory cytokine genes such as TNF-α, IL-12, IL-23, and other proinflammatory cytokines (72–75). In recent findings, TIRAP was found to interact with the AP-1 subunit c-Jun in endotoxin-induced macrophages. This drives the transactivation of c-Jun, its translocation to the nucleus, and a proinflammatory immune response (32). Careful analysis of the molecular docking of a TIRAP-c-Jun crystal structure with immunoprecipitation experiments revealed the direct interaction of TIRAP with c-Jun (32). The pharmacological inhibitor Gefitinib, which abrogates the interaction of TIRAP with c-Jun, specifically reveals the importance of TIRAP-mediated c-Jun transactivation; treatment with Gefitinib drastically reduced the expression of several proinflammatory cytokines (IL-12, IL-23, TNF-α) in both bone-marrow-derived macrophages and in animals (32).

## TIRAP and p38 Mitogen-Activated Protein Kinase

As mentioned above, p38 MAPK is one of three members of the MAPK protein family, that in response to LPS, induces the expression of proinflammatory cytokines (IL-12, IL-23, TNF-α, IL-6, and IL-1β) through activation of the downstream transcription factor AP-1 (31). In addition, the MyD88-dependent TLR4/TLR2 induced activation of MAPKs and NF-κB and subsequent proinflammatory response is well characterized (3, 5, 15, 31, 76). Interestingly, this inflammatory response is also regulated through the direct interaction of TIRAP with p38 MAPK and PKCδ in LPS-stimulated macrophages (30) and suggests that TIRAP has multiple functions in the induction of MAPK signaling.

## TIRAP and Caspase-1

Two reports on the interaction of TIRAP with Caspase-1 have highlighted the importance of this interaction to macrophage signaling events (33, 34). Caspases are evolutionarily conserved enzymes with aspartate-specific, cysteine dependent proteolytic activity, which are involved in inflammation and apoptosis (77, 78). Caspase-1, formerly called IL-1beta converting enzyme (ICE), is synthesized as a zymogen precursor and is cleaved into its p20 (20kDa) and p10 (10kDa) active catalytic subunits and a non-catalytic Caspase Activation and Recruitment Domain (CARD) (79, 80). Active caspase-1, often found in a multi-protein complex called the inflammasome, cleaves the inactive pro-forms of IL-1β and IL-18 to generate active cytokines. This function of caspase-1, as well as activation of gasdermin-D (GSDMD), is critical for the inflammatory immune response of LPS-primed macrophages (80, 81). TIRAP first found to interact with caspase-1 by yeast two-hybrid, is not required for the activation of Caspase-1 (33). Instead, caspase-1 appears to cleave TIRAP (but not MyD88), and this is required for TLR4, and TLR2 mediated NF-κB, p38 MAPK activation, and cytokine production but not IL-1 and TLR7 signaling, which is TIRAP independent (33, 34). After LPS stimulation of macrophages, caspase-1 was reported to cleave after D198 in the C-terminal region of TIRAP. However, the significance of this cleavage was questioned by another study, which found that cleavage was not required for NF-κB activity (34). Instead, it appears that mutation of D198A disrupts TLR4 mediated signaling due to the loss in the acidic amino acid at this site and not due to loss of caspase-1 cleavage. Indeed, TIRAP-D198E remained functional, suggesting that TIRAP is functional in its full-length form (34). Therefore, whether the caspase-1-TIRAP interaction is of biological relevance requires further study.

## TIRAP and Tumor Necrosis Factor Receptor (TNFR)-Associated Factor 6 (TRAF6)

A novel role for TIRAP in NF-κB p65 trans-activation was described after the identification that TIRAP interacts with TRAF-6 (20). The interaction following activation of TLR2 or TLR4 is direct in nature and independent of membrane localization (**Figure 2**). The TRAF family of adaptor proteins and E3 ubiquitin ligases comprises of 6 members, viz., TRAF1, TRAF2, TRAF3, TRAF4, TRAF5, and TRAF6, with each member having distinct functions in the regulation of immune signaling (82). A short motif, Pro-X-Glu-X-X-Z (X: any amino acid; Z: aromatic/acidic residue), acts as a TRAF6 binding motif that is found in various receptors and adaptor proteins (82). TIRAP contains a TRAF6 binding domain, with mutation of this motif (TIRAP-E190A), abolishing interaction with TRAF6 and preventing signal transduction (20). Functionally, the interaction of TIRAP with TRAF6 promotes serine-536 phosphorylation of the p65 subunit of NF-κB, which regulates its transcriptional activation, rather than nuclear translocation (20). In contrast, a recent study based on a mathematical data-model suggests that TIRAP-independent MyD88 activation and Myddosome complex formation in TLR4 signaling does not require TRAF6 (83).

## TIRAP and E3 Ubiquitin-Protein Ligase RNF216 (Triad3A)

Triad3A is a RING finger-type E3 ubiquitin-protein ligase that recognizes and interacts with the TIR domain of TLRs, promoting their proteolytic degradation (22, 42). Specifically, Triad3A causes K48-linked ubiquitination and degradation of TLR4 and TLR9. In addition, Fearns et al. (2006) demonstrated that overexpressed Triad3A directly interacts with and results in degradation of the TLR4 adaptor proteins TIRAP, TRIF, and RIP1 but not other adaptor proteins such as MyD88 and TRAM (22). This suggests that TIRAP is post-translationally regulated *via* different E3 ligases (CLIP170 and Triad3A). Future work should determine the relative contributions and functions to proinflammatory immune signaling.

## TIRAP and Interleukin-1 Receptor-Associated Kinase-Like 2 (IRAK-2)

The interleukin-1 receptor-associated kinase (IRAK) family protein kinase consists of four members, IRAK-1, IRAK-2, IRAK-3/M, and IRAK-4 (84). The sequential activation and recruitment of IRAKs, excluding IRAK-M, in MyD88 dependent signaling is well studied and reported in TLR signaling (76, 85–87). In the TLR-4-TIRAP-MyD88 mydossome complex, active MyD88 interacts with the N- terminal death domains of IRAK4, triggering a series of phosphorylation events and recruitment of IRAK-1 and IRAK-2, which are required for TRAF-6 ubiquitination, activation, and downstream activation of NF-κB (88, 89). In addition, IRAK-2, but not IRAK-1, directly interacts with TIRAP, and this results in NF-κB activation (2, 26). Further evidence for the critical role of IRAK-2 comes from the observation that a dominant-negative variant blocks TIRAP-induced NF-κB activation. The precise residues that mediate the TIRAP-IRAK-2 interaction remain unknown, but the critical tyrosine residues of TIRAP (Y86, Y106, and Y159) are not required (26). Further research is required to understand the precise physiological role of the IRAK-2-TIRAP signaling axis and test whether it represents a means for developing targeted therapeutics that control inflammation.

## TIRAP and Interleukin-1 Receptor-Associated Kinase-Like 1/4 (IRAK1/4)

Interestingly, the interaction of TIRAP with IRAK-4 and IRAK-1 can result in TIRAP degradation following its phosphorylation and ubiquitination, and therefore inhibition of signaling. In this way, IRAK-1/-4 negatively regulates TLR-4/2 signaling by expressing an auto-active IRAK4 inducing TIRAP degradation (35). The interaction appears to be mediated by the TIR domain of TIRAP, with mutation of proline at position 125 (P125H) in the BB loop abolishing the interaction. Both IRAK-4 and IRAK-1 phosphorylate multiple sites, including T28 on TIRAP, that are required for subsequent ubiquitination and degradation (35). Additional research is required to dissect the additional phosphorylation sites within TIRAP, as well as the ubiquitination sites that control TIRAP degradation. Furthermore, the selective role of IRAK-4 and IRAK-1 both as positive and negative regulators is an important consideration in the investigation of inflammation. It is interesting to speculate that over time IRAK-1 and IRAK-4 switch from a positive role in MyD88 dependent inflammatory signaling to a negative one, helping to dampen signaling and prevent too much inflammation. What controls this switch should be investigated using a dose-dependent endotoxin challenge over time, with careful monitoring of TIRAP complex composition.

## TIRAP and Suppressor of Cytokine Signaling 1 (SOCS1)

The suppressor of cytokine signaling (SOCS1) serves as a key physiological regulator of both innate and adaptive immunity acting through macrophages, dendritic cells, B-cells, and T-cells (90). Structurally, the eight intracellular members of the SOCS family, SOCS1-SOCS7 and CIS, have a central SH2 domain, N-terminal extended SH2 subdomain and a variable region, and C-terminal 40 amino acid SOCS box. The SOCS box recruits factors for E3 ubiquitin ligase mediated target protein ubiquitination followed by proteasomal degradation (90, 91). Previous studies described SOCS1 as a negative intracellular regulator in the cytokine-induced JAK-STAT pathway; however, subsequent studies have addressed the role of SOCS1 that is associated with TLR4 and TLR2 signaling as an E3 ligase that polyubiquitinates TIRAP along with other proteins such as p65 and IRAK1, causing their 26S proteasomal degradation (23–25). Analogous to PEST (Proline, Glutamic acid, Serine, and Threonine) motif-containing proteins such as IκB, the PEST region of TIRAP mediates its phosphorylation, lysine polyubiquitination, and degradation, after LPS and Pam_3_Cys induced TLR4 and TLR2 signaling, but not following TLR7 and TLR9. Unlike TIRAP, MyD88 does not contain a PEST domain. This degradation appears dependent on SOCS1; a mutant SOCS1 (SH2 and SOCS Box) variant, as well as SOCS1 deficient macrophages, are unable to target and degrade TIRAP in response to LPS ligation, whereas wild type SOCS1 interacted and degraded TIRAP as early as 15 to 30 minutes post LPS stimulation (23). As tyrosine phosphorylation of TIRAP is required for SOCS1-mediated degradation, it appears that TIRAP is degraded only after BTK has been activated and has phosphorylated TIRAP (23, 24). Further, the tyrosine phosphorylated TIRAP generates a binding site for SH2-domain of SOCS1 (24). Accordingly, SOCS1-mediates the negative regulation of TIRAP to prevent sustained NF-κB and MAPK activities in macrophages (23). In addition to TIRAP phosphorylation, SOCS1 functional activity also depends on NOS1-derived NO production (74, 92). In response to LPS in TLR4 signaling, NOS1 derived NO production resulted in degradation of wild-type SOCS1 post-S-nitrosation, whereas pharmacological inhibition of NOS1 resulted in increased SOCS1 expression and a concomitant increase in TIRAP degradation (74). Therefore, it will be a rewarding effort to elucidate how the NOS1 axis, propagating sustained TIRAP activation, balances with TIRAP degradation mediated *via* BTK and SOCS1 during different inflammatory events (**Figure 2**).

## Computational Prediction of TIRAP Phosphorylation and Nitrosylation Sites

**Figure 3:**
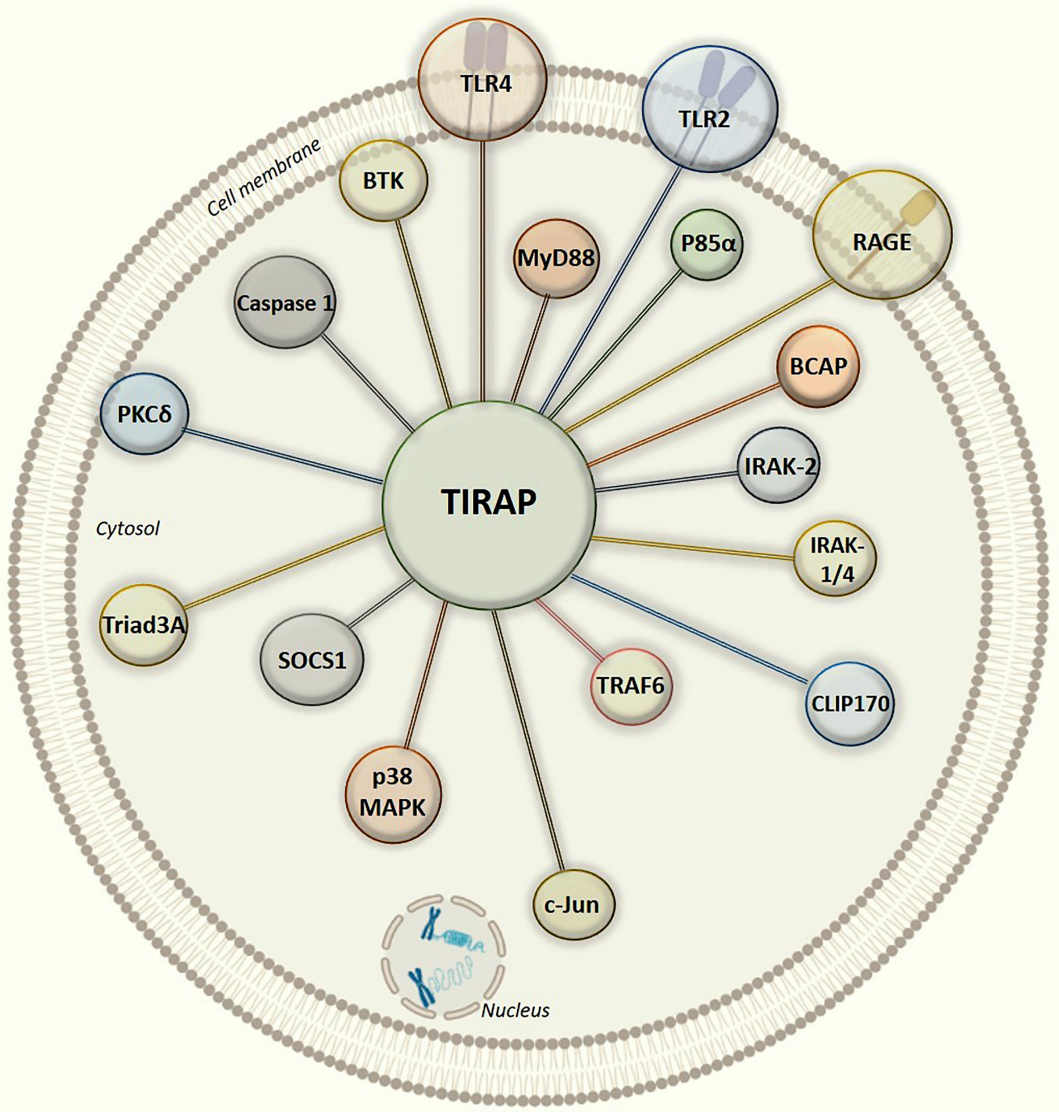
Graphical illustration of intracellular protein-protein interaction network of TIRAP with experimentally defined partner proteins.

Studies have described the role of TIRAP in modifying the innate immune response elicited largely by endotoxin (4, 5, 9, 15, 17, 93). However, the increasing number of TIRAP partner proteins and their role in the activation of inflammatory response (**Figures 2** and **3**) pose great potential as therapeutic targets, demanding further structural elucidation. Here we have attempted to give a brief insight into the basic architecture of TIRAP and its protein-binding domain. The human TIRAP gene encodes a 221 amino acid protein (10). Segregating TIRAP protein sequence gives us an N-terminal region spanning from the 1^st^ to 83^rd^ amino acid containing the PBD (Phosphatidylinositol 4,5-bisphosphate (PIP_2_) Binding Domain) from the 15^th^ to 35^th^ amino acid sequence that is rich in lysine residues. A C-terminal region mainly encodes the TIR Domain, spanning for 130 amino acid long from 84^th^ to 213^th^ (10, 13, 94). Also, we used the Motif search tool at www.genome.jp (https://www.genome.jp/tools/motif/) to obtain the domain details from different databases as produced by the tool search results.

In all the interactions discussed above (**Figure 3**), it is interesting to note that the TIR domain is centrally involved in making interactions with the TIRAP partner proteins. Though, there may be numerous post-translational modifications which can regulate TIRAP interactions, however, the experimental evidence of tyrosine phosphorylation in the TIR domain is one of the most common and validated. Besides, the macrophages largely produce NOS (Nitric oxide synthase) mediated NO (nitric oxide) which has a crucial role in regulating inflammatory responses, for example, NOS1 protects TIRAP degradation from SOCS1 (74, 92). Unlike phosphorylation, there is no experimental evidence of NO mediated nitrosylation sites yet in TIRAP, however, we may presume that the presence of Cystine S-nitrosylation site in TIR domain may be another key factor in regulating the interaction and signaling events. However, S-glutathionylation of cysteine at C91 in TIR domain has been shown to increase the interaction of TIRAP with MyD88 (95).

Besides the known phosphorylation sites, TIR domain might also encompass other sites which might be crucial for a string of TIRAP mediated interactions. Hence, in the current review, we also attempted to computationally predict possible Serine/threonine/tyrosine phosphorylation and S-nitrosylation sites on TIRAP. Briefly, the human TIRAP TIR domain sequence retrieved from UniProt (UniProtKB- P58753) was used for the prediction and study. We searched the protein phosphorylation database PhosphoSitePlus server (https://www.phosphosite.org/homeAction) and the same sites were retrieved for human TIRAP TIR domain. Further, the predictions of possible phosphorylation sites in TIR domain were obtained from kinase-based phosphorylation prediction tools; GPS-phosphorylation (http://gps.biocuckoo.cn/) (96) and NetPhos 3.1 (http://www.cbs.dtu.dk/services/NetPhos/) (97). Including the experimentally proved sites, both the tools predicted same number of a total 21 probable phosphorylation sites (10 serine sites, 05 threonine sites, and 06 tyrosine sites) (**Supplementary Figure 1**). Similarly, the S-Nitrosylation (SNO) sites in TIR domain were predicted from three different computational tools [GPS-SNO (http://sno.biocuckoo.org) (98), DeepNitro (www.deepnitro.renlab.org) (99) and SNOSite (http://csb.cse.yzu.edu.tw/SNOSite/predict.php)] (100). Interestingly, all the SNO sites predicted from these three independent tools were found similar (**Supplementary Figure 1**). The graphical representation of TIRAP and its domains, as presented in **Supplementary Figure 1**, was further depicted using the DOG Domain illustrator tool (http://dog.biocuckoo.org/). In support of these predictions, the tyrosine residues Y86, Y106, Y159, and Y187 have been reported to be the experimentally confirmed phosphorylation sites which modulate the interaction of TIRAP with BTK protein and downstream transcription factors activation (13, 17, 26, 27).

Since the discovery of TIRAP, it has been shown to have an elaborate role in inflammatory signalling. As summarized in this review, many investigators have identified its diverse functions other than that of its role as an adaptor protein. The dynamic nature of TIRAP interaction with several upstream and downstream signaling proteins in varied inflammatory pathways draws immense curiosity towards the post-translational modifications in its TIR domain. Interestingly, a recent study described the interaction of TIRAP and MyD88 with IL1R1 (interleukin-1 receptor like-1) receptor in response to a *Helicobacter pylori* released stimuli (101). Though this study focuses on experimentally validated phosphorylation sites of TIRAP TIR domain, it would be interesting to experimentally identify novel modifications sites on TIRAP which may reveal other crucial interactions involved in signaling pathways.

## Conclusion

The report that TIRAP acts as a second adaptor protein after MyD88 in the year 2001 was marked as a major discovery in the mechanism of TLR4-dependent inflammatory signaling. Subsequently, two more adaptor proteins, TRIF and TRAM, mostly responsible for IRF3 activation, were added to the family of TIR domain-containing adaptor proteins. More recent studies show that TIRAP not only acts as a bridging protein between TLR4/2 and MyD88, but also propagates transduction of downstream signaling events, sometimes in a MyD88-independent manner. Clearly, the ability of TIRAP to interact and collaborate with several signaling molecules in a context-dependent manner means this protein is a major regulator of cell signaling.

## Author Contributions

Conceptualization: MB. Investigation: MB. Writing (Original Draft): MB and SR. Reviewing and editing TT, RI, KW, US, and DL. Supervision: TT. All authors contributed to the article and approved the submitted version.

## Funding

A Biotechnology and Biological Sciences Research Council (BBSRC) David Phillips Fellowship (BB/R011834/1) funds TLMT.

## Conflict of Interest

The authors declare that the research was conducted in the absence of any commercial or financial relationships that could be construed as a potential conflict of interest.

## Supplementary Material

Supplementary Figure 1 Representation of TIRAP TIR (Toll/Interleukin-1 Receptor) domain and computational prediction of serine (S), threonine (T) and tyrosine (Y) phosphorylation and cysteine (C) S-nitrosylation (SNO) positions. The TIRAP protein containing an N-terminal PBD and C-terminal TIR domain are highlighted. The predicted phosphorylation sites on top and SNO sites at bottom of domain map are indicated in blue and light brown colors, respectively. The experimentally proved phosphorylation sites (Y86, Y106, Y159, and Y187) are marked with asterisk. The predicted phosphorylation sites were obtained from kinase-based computational tools GPS-Phosphorylation (http://gps.biocuckoo.cn/) and NetPhos 3.1 (http://www.cbs.dtu.dk/services/NetPhos/). For cysteine (C) SNO sites, computational tools GPS-SNO (http://sno.biocuckoo.org/index.php), DeepNitro (www.deepnitro.renlab.org), and SNOSite (http://csb.cse.yzu.edu.tw/SNOSite/predict.php) were used.
