# Supplementary figures and images for: TIRAP in the Mechanism of Inflammation
Source: Front Immunol. 2021 Jul 8;12:697588. doi: 10.3389/fimmu.2021.697588 (PMC8297548; doi:10.3389/fimmu.2021.697588)

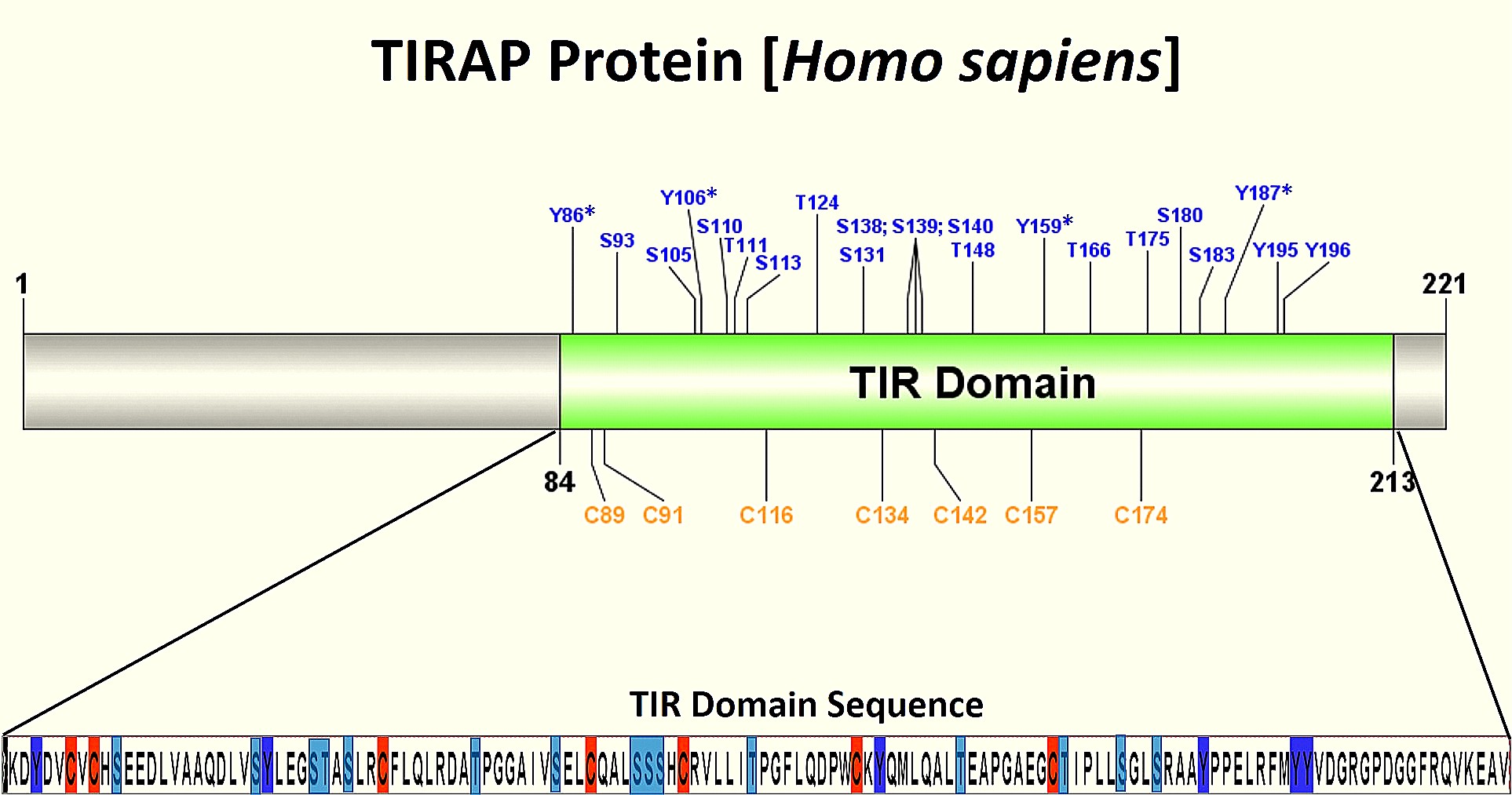

Supplement: Supplementary Figure 1 — Representation of TIRAP TIR (Toll/Interleukin-1 Receptor) domain and computational prediction of serine (S), threonine (T) and tyrosine (Y) phosphorylation and cysteine (C) S-nitrosylation (SNO) positions. The TIRAP protein containing an N-terminal PBD and C-terminal TIR domain are highlighted. The predicted phosphorylation sites on top and SNO sites at bottom of domain map are indicated in blue and light brown colors, respectively. The experimentally proved phosphorylation sites (Y86, Y106, Y159, and Y187) are marked with asterisk. The predicted phosphorylation sites were obtained from kinase-based computational tools GPS-Phosphorylation (http://gps.biocuckoo.cn/) and NetPhos 3.1 (http://www.cbs.dtu.dk/services/NetPhos/). For cysteine (C) SNO sites, computational tools GPS-SNO (http://sno.biocuckoo.org/index.php), DeepNitro (www.deepnitro.renlab.org), and SNOSite (http://csb.cse.yzu.edu.tw/SNOSite/predict.php) were used. [file Image_1.jpg]
